# The Strong Protective Effect of Circumcision against Cancer of the Penis

**DOI:** 10.1155/2011/812368

**Published:** 2011-05-22

**Authors:** Brian J. Morris, Ronald H. Gray, Xavier Castellsague, F. Xavier Bosch, Daniel T. Halperin, Jake H. Waskett, Catherine A. Hankins

**Affiliations:** ^1^School of Medical Sciences and Bosch Institute, Sydney Medical School, The University of Sydney, Sydney, NSW 2006, Australia; ^2^Population and Family Planning, Bloomberg School of Public Health, Johns Hopkins University, Baltimore, MD 21205, USA; ^3^Institut Català d'Oncologia (ICO), IDIBELL, CIBERESP, RTICC, 08908 L'Hospitalet de Llobregat, Catalonia, Spain; ^4^Department of Global Health and Population, Harvard School of Public Health, Boston, MA 02115, USA; ^5^Circumcision Independent Reference and Commentary Service, Radcliffe, Manchester M261JR, UK; ^6^Joint United Nations Programme on HIV/AIDS, 1211 Geneva, Switzerland

## Abstract

Male circumcision protects against cancer of the penis, the invasive form of which is a devastating disease confined almost exclusively to uncircumcised men. Major etiological factors are phimosis, balanitis, and high-risk types of human papillomavirus (HPV), which are more prevalent in the glans penis and coronal sulcus covered by the foreskin, as well as on the penile shaft, of uncircumcised men. Circumcised men clear HPV infections more quickly. Phimosis (a constricted foreskin opening impeding the passage of urine) is confined to uncircumcised men, in whom balanitis (affecting 10%) is more common than in circumcised men. Each is strongly associated with risk of penile cancer. These findings have led to calls for promotion of male circumcision, especially in infancy, to help reduce the global burden of penile cancer. Even more relevant globally is protection from cervical cancer, which is 10-times more common, being much higher in women with uncircumcised male partners. Male circumcision also provides indirect protection against various other infections in women, along with direct protection for men from a number of genital tract infections, including HIV. Given that adverse consequences of medical male circumcision, especially when performed in infancy, are rare, this simple prophylactic procedure should be promoted.

## 1. Introduction

Penile cancer is a devastating disease, although uncommon in developed countries. It accounts for less than 1% of all malignancies in men in the USA and 0.1% of cancer deaths. The 5-year survival rate is approximately 50% [1], having decreased little over recent decades [2, 3]. The disease is confined almost exclusively to men who are uncircumcised, the lifetime risk of penile cancer in an uncircumcised man being 1 in 600 in the USA and 1 in 900 in Denmark [2]. These figures are not to be confused with the often quoted annual incidence figure of the order of 1 in 100,000 [1, 4]. In the USA the annual incidence of primary malignant penile cancer decreased from 0.84 per 100,000 men in 1973 to 0.58 per 100,000 in 2002 [5]. Squamous cell carcinoma is the most common type of penile cancer. In the USA it represents 93% of all penile malignancies [6]. From 1998 to 2003, 4,967 men were diagnosed with invasive squamous cell carcinoma, representing less than 1% of all new cancers in men and occurring at 0.81 cases per 100,000 US men over the five years [7]. In 2010 there were 1,250 new cases of penile cancer and 310 deaths [8]. 

In the USA, Hispanic men have the highest incidence (0.66 per 100,000), then Black men (0.40 per 100,000), White (0.39), American Indians (0.28), and Asian-Pacific Islanders (0.24) [6]. For ages >85 years incidence was 4.7 and 3.6 per 100,000 in Hispanic and Black men, respectively [6]. A decline in incidence by 1.9% per year in Blacks and 1.2% in Whites between 1995 and 2003 has been attributed to earlier detection and treatment [6]. The majority (61%) were diagnoses at the localized stage. Differences were apparent according to geographical region. Other figures, published in 2007, give annual incidence figures of 1.01 per 100,000 for white Hispanics, 0.77 for Alaskan native/American Indians, 0.62 per 100,000 for Blacks and 0.51 for non-Hispanic whites [5]. These figures correlate inversely with incidence of male circumcision in these groups.

## 2. Lack of Circumcision as a Major Risk Factor for Penile Cancer

An extensive review in 2006 concluded that penile cancer is an “emerging problem”, noting that “public health measures, such as prophylactic use of circumcision, have proven successful” [10]. Neonatal circumcision virtually abolishes the risk [11]. 

The penile cancer incidence data for the USA have to be viewed in the context of the high proportion of circumcised men, especially in older age groups, and the age group affected, where mean age at presentation is 60 years [12]. Thus an incidence of approximately 1 in 100,000 males per year of life translates to 75 in 100,000 during each man's lifetime (assuming an average life expectancy of 75 years). However, when one considers that penile cancer occurs almost entirely in uncircumcised men, by assuming that these represent 30% of males in the USA, the chance of an uncircumcised man getting penile cancer would be 75 per 30,000, that is, 1 in 400, which accords with the lifetime risk noted above [2].

In five major series in the USA, starting in 1932 [13], not one man with invasive penile cancer had been circumcised neonatally [3]. Another report noted 50,000 cases of penile cancer in the USA from 1930 to 1990, resulting in 10,000 deaths [14]. Only 10 of the cases occurred in circumcised men, but all of these men had been circumcised later in life. Penile cancer is in fact so rare in a man circumcised in infancy, that when it does occur it can be the subject of a published case report [15]. The finite residual risk is greater in those circumcised after the newborn period but is still less than for men who are not circumcised [16]. In Saudi Arabia, where circumcision is performed in older children, penile cancer in circumcised men (average age 62) was associated with ritual, nonclassical, so-called “vigorous”, circumcision [17]. 

In circumcised men, the very low lifetime risk has been estimated as 1 in 50,000 to 1 in 12,000,000 [18, 19]. For 213 cases in California only 2 of 89 men with invasive penile cancer had been circumcised in infancy, so these authors concluded that uncircumcised men had a 22-fold higher risk [20, 21]. Of 118 patients with the localized, and thus more easily curable, variety of penile cancer—carcinoma *in situ* (which is not lethal)—only 16 had been circumcised as newborns, that is, incidence was 7.3-fold higher in the uncircumcised [3, 20, 21]. A study in Louisiana found that only 2 of 45 penile cancer patients had been circumcised in infancy [22]. 

In Denmark, where circumcision prevalence is 2%, penile cancer has nevertheless been decreasing steadily [23] in parallel with an increase in indoor bathrooms, consistent with improved hygiene as a possible factor. Urban unmarried Danish men were more likely to develop cancers. Since the rate of penile cancer in Denmark is slightly lower than in the USA other factors besides circumcision would appear to be relevant, be they diet, lifestyle, climate or other. The statistics for Denmark have been used by opponents of circumcision to draw a very tenuous conclusion that lack of circumcision is not associated with penile cancer. The Danes themselves have concluded that although their uncircumcised men might appear to be at slightly lower risk, this is only 1 in 900 as opposed to 1 in 600 in the USA [2]. A study in Spain concluded that “circumcision should be performed in childhood [as a] prophylactic [to penile cancer]” [24].

As a historical point of interest, Diego Rivera, the famous Mexican muralist, who was renowned for having multiple sexual partners in a country where most men are uncircumcised, developed penile cancer [25]. He refused penectomy, instead travelling to the Soviet Union for radiation therapy, and died a painful death from the disease and the side effects of his therapy.

In Australia, cases averaged 66 per year over the decade to 2003 [26]. Typical age distribution of cases was 4% for men in their 30s, 14% in their 40s, 15% in their 50s, 22% in their 60s, 31% in their 70s, and 12% aged over 80 [27]. One in 4 died as a result, the death rate being higher in older men. The annual incidence of penile cancer was 0.8 per 100,000 population [27], that is, similar to the US figures above, and was also similar in each state of Australia. Life-time (age 0–74) risk was estimated as 1 in 1,574 males [27]. As in the USA, over two-thirds of older men in Australia are circumcised, so the decline in the proportion of uncircumcised males in the Australian population, that occurred when infant male circumcision dropped precipitously in the 1970s, would, by itself, be expected to be accompanied by a rise in the incidence of penile cancer.

In Israel, where almost all males are circumcised, the rate of penile cancer is extremely low: 0.1 per 100,000, that is, is 1/10th that of Denmark [28, 29].

Low- and middle-income countries have a much higher incidence of penile cancer: approximately 3–10 cases per 100,000 per year [2]. In countries where circumcision is not practiced routinely, such as those in South America and parts of Africa, penile cancer can be ten times more common than in high-income countries, representing 10–22% of all male cancers [1, 30, 31]. In Uganda and some other African countries it is the most common malignancy in males, leading to calls for more male circumcision [32]. Enormous differences are, moreover, seen amongst low- and middle-income nations corresponding to differences in circumcision prevalence in each country or ethnic subgroup. In Puerto Rico [29], India, and Brazil [33, 34], where most men are uncircumcised, penile cancer is quite common. Brazil has one of the highest rates of penile cancer, 6–14 per 100,000 males per year, comprising 2–6% of all male neoplasias, with 7% of cases occurring in men aged under 35, and 39% in men older than 66 [33]. Among cases, 87% are uncircumcised. All tumors seen in men circumcised in childhood were of low grade, whereas 12% of those circumcised in adulthood had high-grade tumors [33]. In at least two Brazilian States (Maranhao and Pernambuco) penile cancer is reportedly the 2nd highest cause of carcinoma death in men (after lung cancer). At the main oncology hospital in Recife, Pernambuco (Brazil's 4th largest city), on average one or two men each week need to have a penile amputation due to this cancer, with prognosis very poor. Many years ago the directors of this hospital were interested in starting a male circumcision promotion program (D.T. Halperin, personal communication).

A statement made in 1973 that “despite overwhelming evidence from urological surgeons that neoplasm of the penis is a lethal disease that can be prevented by removal of the foreskin, some physicians continue to argue against routine newborn circumcision in a highly emotional fashion” [35] is just as true today. In the interests of public health, such denial of evidence needs to be successfully confronted and countered.

## 3. The Role of Human Papillomavirus (HPV) Infection in Etiology of Penile Cancer

### 3.1. Overview

Cancer of the penis can present as carcinoma *in situ* or invasive penile cancer. In the USA the proportion of each of these is similar, 45% and 55%, respectively. Invasive penile cancer is lethal, whereas carcinoma *in situ* is comparatively benign. The former is not necessarily a continuum of the latter [36]. 

HPV is present in most basaloid and warty carcinomas which comprise 50% of cases [30]. Similarly, in women, half of all vulvar carcinomas are HPV positive. In contrast, virtually all cervical cancers are positive for high-risk HPVs. High-risk HPV is found more frequently in verrucous carcinomas than giant condylomas (which are caused by low-risk HPV). Although relatively harmless, such benign condylomas are readily apparent and can be quite confronting in appearance. Keratinizing and verrucous carcinomas are HPV positive in one-third of cases [30]. A Spanish study found HPV in 78% of penile carcinoma specimens, with 84% of these containing the most common high-risk type, HPV16, and 11% having the second most common high-risk type, HPV18 [37]. A Danish study found 65% of squamous cell carcinomas had HPV, with 92% of these being HPV16 [38]. In Thailand, HPV was found in 82% of penile cancers, of which 55% had HPV18, 43% had the low-risk type HPV6, and a large proportion had both [39].

**Figure 1:**
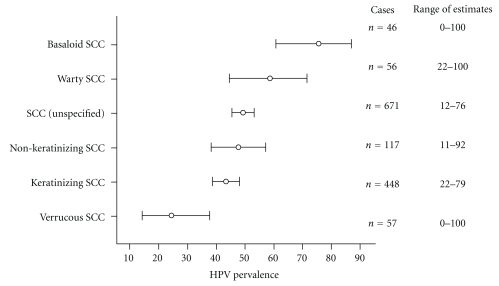
Prevalence of HPV in different histological types of squamous cell carcinoma of the penis. Bars indicate 95% confidence intervals. Modified from Miralles-Guri et al. [9].

In a review of 31 studies, representing 1466 penile carcinomas, overall prevalence was 46.9% [9]. Of those positive for HPV, prevalence of the different types was HPV16 (60.2%), HPV18 (13.4%), HPV6/11 (8.1%), HPV31 (1.2%), HPV45 (1.2%), HPV33 (1.0%), HPV52 (0.6%), and other types 2.5%. The most frequent HPV-related histological types were basaloid and warty squamous cell carcinomas.  Figure 1 shows the prevalence of HPV in these and other types of squamous cell carcinoma.

There is good reason to suspect that the high-risk HPV types (16, 18, and numerous rarer types) found in a large proportion of cases, are involved in the causation of many penile cancers [40], since they are the same viral types as are responsible for virtually all cases of cervical cancer in women (see below). 

High-risk HPV types produce flat warts that are normally only visible by application of dilute acetic acid (e.g., vinegar) to the penis. The majority of HPV infections are subclinical; moreover, HPV infection is more prevalent in uncircumcised men having balanoposthitis [41]. High-risk HPV prevalence data should not be confused with genital warts incidence figures. Genital warts are large and readily visible, and are caused by the relatively benign HPV types 6 and 11 [42].

### 3.2. Circumcision Protects against HPV Infection

There have been numerous studies comparing HPV prevalence in circumcised and uncircumcised men in different countries, racial groups, and ages [7, 41, 43–58] (Table 1).

A large multinational study published in the *New England Journal of Medicine* in 2002 detected HPV in 19.6% of 847 uncircumcised men, compared to only 5.5% of 292 circumcised men (overall odds ratio (OR) after adjusting for potential confounding factors = 0.37; 95% confidence intervals (CI) = 0.16–0.85; *P* < .001) [45] (Table 1(a)). (All odds ratios cited in this paper are significant at the *P* = .05 level unless otherwise indicated.) In this study, samples were collected from the urethra and glans penis/coronal sulcus. A study at an STI clinic in Copenhagen, Denmark, found that being uncircumcised was associated with a 5-fold higher likelihood of being infected with HPV [54] (Table 1(b)). Among STI clinic attendees in the USA, HPV was 1.5 times higher in uncircumcised men [44] (Table 1(c)). In Mexico, men attending vasectomy clinics had 5 times higher HPV if they were uncircumcised [57] (Table 1(g)). Another Mexican study, involving healthy military men, found a 10-fold higher OR for persistent HPV infection in uncircumcised men [48] (Table 1(f)). In the HIM study, involving men in the USA, Mexico, and Brazil, high-risk HPV types were lower in circumcised men (OR 0.70) as were low-risk HPV types (OR 0.63) [46] (Table 1(k)).

### 3.3. Meta-Analyses

A meta-analysis of 8 studies published in 2007 found an association of circumcision with a statistically significant reduced risk of penile HPV and related lesions (OR = 0.56; 95% CI = 0.39–0.82) [60]. The meta-analysis was prompted by the publication of a “biased, inaccurate and misleading meta-analysis” by Van Howe [61]. Other meta-analyses of circumcision and STIs by Van Howe [62, 63] have similarly been shown by experts in the field to be fundamentally flawed [64–66] and one [63] was even shown to contain false source data [66], thus accounting for its surprising conclusion. 

**Figure 2:**
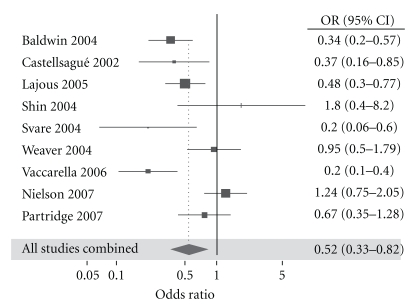
Difference in prevalence of high-risk HPV types between circumcised and uncircumcised men.

A subsequent meta-analysis, published in 2009, examined high-risk HPV types in 14 studies (5 US, 2 Mexican, 2 Australian and one each from England, Denmark, South Korea, Kenya, and the multinational study in 2002 referred to above). It assessed data for 5,880 circumcised men and 4,257 uncircumcised men, finding circumcision to be protective, the OR for HPV infection being 0.52 (95% CI = 0.33–0.82) [67] (Figure 2). This meta-analysis found a marginally lower prevalence of low-risk HPV types in circumcised men, although this was not statistically significant (OR 0.89; 95% CI 0.59–1.33). This is likely because low-risk HPV types are associated with visible warts that tend to occur on the shaft of the penis, a site of infection unlikely to be affected by circumcision [67].

### 3.4. Distribution of HPV on Penis

**Figure 3:**
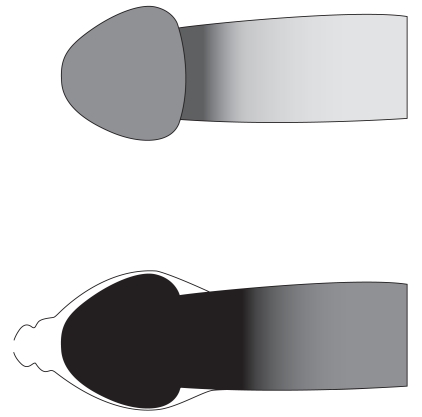
The circumcised and uncircumcised penis, depicting the differences in prevalence of HPV between each.

High-risk HPV types exhibit a much higher prevalence with proximity to the tip of the penis (Figure 3). In an early study, the distribution of HPV was reported as 28% foreskin, 24% shaft, 17% scrotum, 16% glans, and 6% urine [58] (Table 1(d)). In another study, HPV prevalence ranged from 41% on the shaft to 4.7% in semen [49] (Table 1(j)). The strength of the association between circumcision and reduced HPV decreased with distance from the prepuce/urethra, with the adjusted OR being 0.17 for the urethra, 0.44 for the glans/corona, and 0.53 for the shaft, with no significant difference found for the scrotum, perianal area, anal canal and semen [49] (Table 1(j)). A study in Hawaii of men who were primarily heterosexual found HPV infection of the glans/coronal sulcus to be 46% in uncircumcised men compared with 29% in circumcised men [59] (Table 1(i)). This study also found that uncircumcised men had a significantly higher risk of oncogenic HPV types (adjusted OR 2.51) and infection with multiple HPV types (adjusted OR 3.56). In uncircumcised men, HPV prevalence on the foreskin (44%) was comparable to that on the glans/coronal sulcus. A study of 2,705 uncircumcised men aged 17–28 in Kisumu, Kenya found high-risk HPV prevalence to be 31.2% on the glans and 12.3% on the shaft (*P* < .0001) [53]. In Amsterdam, HPV16 was the most common type, with 29% infected with more than one type. Not surprisingly, men with HPV were also more likely to have other STIs. Only 1% of men had visible genital warts. A randomized controlled trial (RCT) has yielded similar findings, as will be presented below.

### 3.5. Randomized Controlled Trials

The protection afforded by circumcision against HPV prevalence on the penis is supported by RCTs in two localities. One of these, conducted in Uganda and published in the *New England Journal of Medicine*, found that 24 months after circumcision the prevalence of high-risk HPV in swabs from the coronal sulcus of the penis was 18% compared to 28% in the uncircumcised men in the control arm of the trial, giving an adjusted prevalence ratio of 0.65, indicative of a 35% protective effect of circumcision [55] (Table 1(m)). When confining the analysis to samples positive for *β*-globin (meaning cellular DNA was present), HPV was found in 14.9% of the circumcised group compared with 26.5% of the uncircumcised group, pointing to a 44% protective effect [55]. Multiple high-risk HPV types were detected in 4.3% of circumcised men and 12.2% of uncircumcised men, indicating a 65% protective effect of circumcision against these [55]. The prevalence of non-high-risk HPV types was 26% versus 39%, in circumcised and uncircumcised, respectively, indicating a protective effect of 35% [55]. These researchers later reported data for the shaft in which they only included swabs that were positive for high-risk HPV by the Roche Linear array assay or for *β*-globin DNA. At 12 months, high-risk HPV was present in 15.5% of shaft samples from 121 circumcised men and 23.8% of 171 uncircumcised men (prevalence risk ratio (PRR) = 0.65), indicating 35% protection [56] (Table 1(o)). Multiple HPV types were found on the shaft of 1.7% of circumcised and 3.8% of uncircumcised men (PRR = 0.45). For the coronal sulcus these values were 21.5% and 36.3%  (PRR = 0.59) for any high-risk HPV type in the circumcised and uncircumcised arms of the trial, and 7.4% and 10.5%  (PRR = 0.71) for multiple high-risk HPV types. HPV was therefore detected more frequently on the coronal sulcus than the shaft.

In another report from the Rakai trials, among 230 circumcised men, 14% acquired new HPV infections over 24 months, compared to 25% of 267 uncircumcised men, giving an adjusted incidence rate ratio of 0.58, meaning a 42% protective effect [68]. The acquisition of multiple high-risk HPV types was 6.7 cases per 100 person years in the intervention arm and 14.8 cases per 100 person-years in the control arm [47] (Table 1(n)). The protective effect was similar for all HPV types. In men who were HIV-positive, the Rakai trial found that multiple new HPV types, both low-risk and high-risk, were acquired in 9.9% of intervention arm subjects and 24.7% of control arm subjects (relative risk (RR) = 0.40; *P* = .01) [69]. The incidence of multiple high-risk HPV infections was reduced significantly in HIV-negative (RR = 0.45) and HIV-positive (RR = 0.53) men [70]. Statistical modeling which accounted for complex correlation within individuals and between HPV genotypes has shown that the current as-treated efficacy of male circumcision for prevention of high-risk HPV infections is greater than the originally reported efficacy which used the individual participant as the unit of analysis [71]. The absence of statistical significance of sex frequency and condom use in the multivariate model implied that partner's HPV carrier status was the fundamental determinant of HPV incidence observed in the men studied.

In another RCT, in South Africa, high-risk HPV in urethral swabs was 34% lower in the circumcised group at 21 months after surgery [43] (Table 1(l)). The authors stated, moreover, that owing to the fact that some men would have already been infected with HPV before inclusion in the trial, the actual effect of circumcision on incident HPV could have been greater than the reduction reported. Others have pointed out that HPV detection may have been less than optimal owing to sampling at the urethra rather than the glans, coronal sulcus, or shaft, so underestimating the efficacy of circumcision in reducing HPV [72, 73]. Being positive for HIV was associated with infection by multiple HPV types (OR = 4.0) [74]. 

The South African RCT found that HIV infection was higher in men positive for high-risk HPV (adjusted incidence rate ratio (IRR) = 3.8; *P* < .001), an association that suggested that high-risk HPV could facilitate HIV acquisition [75]. However, confounding by sexual behavior and concurrent transmission of each virus is possible, meaning that the validity of this assertion remains uncertain. Circumcision reduced low-risk HPV infections in both HIV-negative and HIV-positive men [75]. In the Kenyan RCT, after controlling for baseline herpes simplex virus-2 serostatus, as well as sexual and sociodemographic status, the hazard ratio for HIV infection among men positive for HPV in glans/coronal sulcus specimens was 1.8 compared with men negative for HPV in such specimens (*P* = .03) [76].

### 3.6. Why HPV Is Higher in Uncircumcised Men

In uncircumcised men, the moist subpreputial space likely provides a more hospitable environment for infection by viruses than the drier environment of the penis lacking a foreskin [77–79]. In women the genital tract can provide a site that acts as a reservoir for high-risk HPV infection at other anatomical sites in the woman [80]. Thus circumcision should reduce autoinfection of other sites in men too, one being the shaft, so explaining the lower infection in the shaft of circumcised men. High-risk HPV replicates in basal epithelial cells of the epidermis [81]. The inner mucosa of the foreskin is only lightly keratinized [78, 82, 83]. Earlier discrepancies in the findings on keratinization most likely resulted from differences in how foreskin tissue was handled and processed subsequent to its excision [84]. The lower keratinization of the foreskin may facilitate access of high-risk HPV to underlying epithelial cells in uncircumcised men. After circumcision, the keratinization of the surgical scar and surrounding tissue would help reduce such epithelial infection. These salient features of the uncircumcised and circumcised penis may help explain why high-risk HPV infection is lower in both the coronal sulcus and the shaft of the circumcised penis.

### 3.7. Circumcised Men Clear HPV Faster

Although HPV seroprevalence was found to be similar in circumcised and uncircumcised men in a longitudinal study in New Zealand [85], indicating similar exposure, the penile prevalence was lower in circumcised men. The explanation is that circumcised men eliminate the infection faster, with serostatus reflecting previous infection in some cases. In support of this, a longitudinal study in Tucson, Arizona, of 285 men aged 18–44 found that circumcised men clear penile oncogenic, but not nononcogenic, HPV infections 6 times faster than do uncircumcised men [86]. In Hawaii, 357 men of average age 29 years, 19% of whom were uncircumcised and 75% of whom were heterosexual with an average of 6.5 prior female sex partners, were tested for HPV types every 2 months for 14 months [87]. Although there was no difference in acquisition of HPV, the clearance of HPV, including that of oncogenic types, from the glans/coronal sulcus took 3 months in the men who were circumcised, compared with 5 months for those who were not (*P* = .04). There was no difference for the shaft or scrotum. In the RCT in Rakai, Uganda, clearance of pre-existing HPV was higher in circumcised men at 216 cases per 100 person years in the intervention arm compared to 159 cases per 100 person-years in the control arm—adjusted RR = 1.39 [47]. 

The ability of circumcised men to clear high-risk HPV faster would further explain their lower risk of penile cancer, and of cervical cancer in their female partner(s). Moreover, as mentioned above, in healthy Mexican military men, OR for persistent HPV infection was 10-times higher in those who were not circumcised [48]. Interestingly, men who had had 16 or more lifetime sex partners were 4.9 times more likely to clear oncogenic HPV infection than men with fewer partners, possibly because of acquired immunity [86].

### 3.8. Vaccination of Males against High-Risk HPV Is Not the Ideal Solution

Female-to-male transmission of HPV involves cervix to penis transmission most frequently, with the glans being most vulnerable [88]. The risk of transmission from the cervix to the penis is 17% per month of exposure, compared with 5% for transmission from the penis to the cervix. After clearance of the virus in one member of the dyad, reinfection in the couple can occur. In a study of 14 high-risk HPV types, resistance to infection was lost at a rate of 1–5% per year the older the subjects became [89]. 

High transmission potential with a low impact on herd immunity means extensive vaccination would be required to substantially reduce the incidence of cancer of the cervix and penis caused by high-risk HPV types [89]. Further, vaccination of males against HPV appears to represent an expensive, inefficient measure for prevention of penile cancer [90], particularly when one considers that high-risk HPV is present in only half of penile cancers. On the other hand, lack of circumcision is a risk factor for phimosis and balanitis (see below) which themselves are risk factors for penile cancer. This would explain why invasive penile cancer is rare in circumcised men, rather than being merely half as common as one might predict based on just the single, but important, risk factor of high-risk HPV [91]. HPV vaccination of males should nevertheless help reduce cervical, anal and perhaps oropharyngeal cancers.

The International Consultation on Penile Cancer determined in November 2008 that the factors associated with invasive penile cancers were high-risk HPV infection (level of evidence 3a–4), phimosis (level of evidence 3a), and balanitis (3a) [92]. In the same issue of *Urology*, the 2009 International Consultation on Urologic Disease Consensus Publishing Group pointed to the well-established role of HPV subtypes in the etiology of cancer of the penis and suggested circumcision and early treatment of phimosis, together with significant changes in global health policy, in addressing this problem [93].

### 3.9. Penile Intraepithelial Neoplasia (PIN) and Cervical Intraepithelial Neoplasia (CIN)

Interestingly, 93% of men whose female partner was positive for early signs of cervical cancer by having CIN had the male equivalent, PIN [94]. This underscores the sexual transmission of high-risk HPV associated with cancer. Oncogenic HPV was present in 75% of patients with PIN grade I, 93% with PIN grade II, and 100% with PIN grade III, the step short of penile cancer [94]. Moreover, the rate of PIN was 10% in uncircumcised men compared with 6% in circumcised men [94]. HPV DNA was found in 80% of tumor specimens, with 69% of these being the high-risk type 16 [36]. Condom use may lower HPV infection as was reported in a study of 393 men in Tucson, Arizona [44]. In another study of 463 men in Tucson and Tampa, condom use halved the prevalence of oncogenic HPV [95]. It is therefore important to note that condom use reduces HPV infection only partially. In the multinational study, although high-risk HPV was lower in condom users, this did not reach statistical significance [45].

## 4. Phimosis

Phimosis is strongly associated with invasive penile carcinoma, the adjusted OR for this being 16 in one study [16] and 11 in another [36]. In fact 45–85% of men with penile cancer have a history of phimosis [16, 33, 96]. Phimosis causes dysplastic (pre-cancerous) changes in the skin of the preputial sac [97]. Although length of the foreskin has been suggested as a factor, the evidence for this is weak [98]. In this study, 52% of penile cancer cases with a long foreskin had phimosis. These findings have led to the conclusion that circumcision in early childhood, by eliminating phimosis, may help prevent penile cancer [36]. A meta-analysis yielded an overall OR of 12.1 (95% CI = 5.6–26.2) (Table 2) for the association of phimosis with penile cancer.

## 5. Smegma

Smegma is a whitish film found under the foreskin of uncircumcised males. It contains bacteria, other microorganisms, dead skin cells, mucous, and other components. Evidence for a role of smegma in the etiology of penile cancer was obtained in an early study [102]. The carcinogenicity of smegma was subsequently confirmed by others [103–105]. It was not clear in these studies from the 1950s and 1960s what component was responsible, but in hindsight it could have been the presence of HPV. Smegma may cause chronic inflammation and recurrent infections that lead to preputial adhesions and phimosis [16, 97]. Male horses produce large amounts of smegma and 23% of cancers in these animals are of the penis. Geldings do not get erections that would normally help eliminate smegma, and in such horses penile cancer is 10 times higher than in stallions [106]. In a meta-analysis of the available data we found an OR of 3.04 (95% CI = 1.29–7.16) for the association between penile cancer and smegma (Table 3).

## 6. Balanitis and Lichen Sclerosis

These conditions are all more prevalent in uncircumcised men. Chronic relapsing balanitis of bacterial, mycotic, or viral origin increases the risk of invasive penile cancer [107, 108]. A history of balanitis has been reported in 45% of penile cancer patients compared with 8% of controls [20, 96]. Penile lichen sclerosis (also termed balanitis xerotica obliterans (BXO)), an inflammatory disorder that can lead to meatal stenosis or phimosis, is associated with penile cancer (reviewed in [10]). BXO is well known in boys where it is more common than is generally assumed [109]. In penile carcinoma patients incidence of lichen sclerosis was initially estimated as 2.6–5.8%, but subsequent research found the rate to be very much higher. In one study it was 28%, with 77% of patients having squamous cell carcinoma and 23% carcinoma *in situ* [110]. Other studies found BXO in 33% [111], 44% [112], and 50% [113] of cases of squamous cell carcinoma. HPV infection was 2.6 times higher amongst patients with penile lichen sclerosis [114]. Lichen sclerosis is not always associated with presence of HPV and it could be that lichen sclerosis acts as a catalyst in the onset of penile cancer [115]. Although this and other evidence supports the view that oncogenic HPV is more prevalent in patients with genital lichen sclerosis (17% versus 9%), other data suggests that lichen sclerosis is a preneoplastic condition unrelated to HPV infection (reviewed in [10]). One review suggested that approximately half of penile squamous cell carcinomas (which represent 95% of penile neoplasms) are associated with lichen sclerosis and half with HPV [91]. A meta-analysis indicates an OR of 3.82 (95% CI = 1.61–9.06) for the association of balanitis with penile cancer (Table 4).

## 7. Herpes, Poor Hygiene, and Other Risk Factors

A cocarcinogenic role of recurrent HSV-2 in penile cancer has also been suggested [116, 117].

The widely used vaginal spermicide, nonoxynol-9, which is abrasive, greatly increases susceptibility of the genital epithelium to HPV16 infection [118]. The vegan alternative to gelatin, carrageenan, a polysaccharide from red seaweed that is a constituent of some vaginal lubricants, was shown to prevent HPV16 infection in mice, and a clinical trial found that it offered women 47% protection against infection by high-risk HPV when used consistently [119]. 

In addition, other factors, such as smoking (4.5-fold increase in risk [36]), poor hygiene (even in the absence of phimosis), and the presence of other STIs have been suspected as contributing to penile cancer as well [3, 120], but it would seem that lack of circumcision is the primary prerequisite, with such other factors adding to the risk in uncircumcised men. Indeed, there is no scientific evidence that improved penile hygiene is effective in reducing the risk of penile cancer in an uncircumcised man [121], although this factor cannot be ruled out. A case-control study in California found no correlation between penile cancer and frequency of bathing or method of cleaning the anogenital area before or after sexual intercourse [16]. 

It therefore seems there may be two etiologic routes to penile cancer: one via sexual transmission of oncogenic HPV in younger men and the other, unrelated to HPV, that mostly affects older men (reviewed in [10]). In each case, lack of circumcision is an important precondition and major risk factor.

## 8. Prostate Cancer

Risk of prostate cancer correlates with a history of STIs, most consistently syphilis, gonorrhoea, Chlamydia, and HPV [122–129]. In contrast to penile cancer, however, no consistent association has been seen between rate of prostate cancer and rate of cervical cancer in different geographic localities [130]. A study of 20,243 men in Finland found infection with HPV18 was associated with a 2.6-fold increase in risk of prostate cancer (*P* < .005) [131]. For HPV16 the increased risk was 2.4-fold. These figures are similar to the increased prevalence of penile HPV infection in uncircumcised men [45]. In contrast, a Swedish study found an association of HPV33, but not HPV16 or HPV18, with prostate cancer [132]. A study in Crete, however, found HPV in only 5% of samples, none of which had the common high-risk types 16 and 18, making a role for HPV unlikely [133]. Consistent with this, a study in Saudi Arabia was unable to detect HPV in any of the prostate biopsies of 56 patients with benign prostatic hyperplasia or prostate cancer [134]. 

The moloney murine leukemia virus homologue known as xenotropic murine leukemia virus (XMRV) (gene: HPC1) was implicated in prostate cancer, initially in patients homozygous for a genetic variant of HPC1 that encodes RNase L, an important component of antiviral defence mechanisms [135]. In a USA study of 334 consecutive prostate resection specimens, DNA for XMRV was found in 6% and XMRV protein expression was found in 23% [136]. This retrovirus was found primarily in malignant epithelial cells, consistent with a role in tumorigenesis and tumor aggressiveness. Its presence in that study was, moreover, independent of polymorphism in the RNase L gene. Others have found an element in the XMRV promoter that causes a doubling of transcription of this gene in response to androgens [137]. XMRV replicates more efficiently in prostate cancer cells due in part to the transcriptional environment [138]. A research team in Berlin, however, failed to find XMRV by PCR in 589 prostate cancers [139]. It is early days, and as yet there is no clear evidence linking XMVR to prostate cancer [140].

The polyomavirus BKV has been found in 19% of cases of prostate cancer in Crete, leading to a suggestion that it could play a role in some of these [133].

*Trichomonas vaginalis*, the most common bacterial STI, was positively correlated with risk of prostate cancer later in life in the US Physicians Health Study [141]. This study measured antibodies to *T. vaginalis* in samples collected a decade before prostate cancer was diagnosed. Seropositivity was associated with a 2-fold increased risk for advanced prostate cancer and a 3-fold higher risk for prostate cancer leading to death. Most men who have *T. vaginalis* infection do not have symptoms. An RCT has found that circumcision can protect against *T. vaginalis* infection, this organism being 46% lower in the men who had been circumcised [142]. An as-treated analysis found *T. vaginalis* to be even lower, 51%, the adjusted OR being 0.41 (*P* = .030).

Such infections may establish a state chronic active inflammation in the prostate, which is associated with a variety of cancers [122]. The rate of STIs has risen over the past decade in many developed countries (e.g., in the UK there are approximately 700,000 cases per year, one-third being in London [143]), suggesting that an increased incidence of prostate cancer may follow. 

Uncircumcised men have a 1.6- to 2.0-fold higher incidence of prostate cancer compared with circumcised men [144–146], and prostate cancer is rare amongst Jews [147]. In Southern California the reduction in risk in circumcised men was 0.5 in whites and 0.6 in blacks [128]. Similarly, in Sweden, uncircumcised males had twice the risk [144]. Of men operated on for prostatic obstruction, only 1.8% of obstructions were cancerous in Jews (circumcised), compared with 19% in non-Jews [146]. A study in the UK in 1996 found an OR for the reduction in risk of prostate cancer in circumcised men of 0.62 [145]. Circumcision prevalence shows an inverse correlation with prostate cancer incidence in 51 countries (*P* = .022), supporting the possibility of circumcision having a protective effect against this cancer (J. H. Waskett, unpublished).

Ascending passage of a particular STI to the prostate could be a causative factor in prostate cancer. An extended clinical trial of the role played by circumcision in the prevention of prostate cancer is needed [148], but this is likely to be a long study.

In the USA, 1 in 6 men develop/get prostate cancer during their lifetime [1]. Annual cases in 2006 were 0.25 million [149] with an average age of diagnosis of 70 years [150]. The circumcision prevalence among these men (born from 1933 to 1947) is approximately 60% [149]. Across the range of a 1.6–2.0-fold increase in risk, calculations show that there are 24–40% (45,000–67,000) more prostate cancer cases than would otherwise be the case if all men were circumcised [151]. 

Treatment by radical prostatectomy leads to shortening in length of the penis by an average of 1.3 cm for the flaccid penis and 2.3 cm (one inch) for the stretched penis, although this generally resolves about a year after surgery [152].

A simple cost-benefit analysis for the USA [151] considered an average cost for radiation therapy of US$13,823 [153] and a combined cost for terminal care of $24,660 per patient for the 41,000 who die of prostate cancer each year [154]. Based on these figures and those above, lack of circumcision was estimated to add $0.8–1.6 billion to the costs of treatment and terminal care each year in the USA [151]. This can be compared with the total for physician and hospital costs for neonatal circumcision in the USA of $195 per infant or a total of $390 million per year [154]. Such a comparison did not take into account indirect costs or the contribution of prostate cancer to disability years of life lost (DALYs).

## 9. The Risk to Women from Sexual Transmission of High-Risk HPV

Any discussion of penile cancer in men cannot fail to mention cervical cancer in women. Sexual transmission of high-risk HPV infection is responsible for virtually all cervical cancer. The incidence of cervical cancer is 10 times higher than that of penile cancer, with 12,000 new cases and 4,000 deaths from cervical cancer each year in the USA [155]. Australian data indicate 725 cases in 2003 (incidence 9.1 per 100,000) and 212 deaths [156]. In the USA, high-risk HPVs account for the loss of 3.3 million DALYs through cervical cancer [157]. The cost of treating cervical disease in the USA each year is approximately $3.5 billion [158]. This figure does not portray the social cost of cervical cancer to individuals and families. 

The study in Denmark referred to earlier that found 5-fold lower HPV in circumcised men concluded that “the female partners of circumcised men are less exposed to cervical cancer because these men are less likely to be infected with HPV” [54].

High-risk HPV types 16, 18 and over a dozen other less common types are responsible for virtually every case of cervical cancer [159–161] and are the same high-risk HPVs that cause PIN, which is the precursor to penile cancer and is the male equivalent of CIN, more often referred to these days as “squamous intra-epithelial lesion” (SIL), the precursor to cervical cancer. Women with cervical cancer are more likely to have partners with PIN [162]. In women with CIN, PIN was present in the male partner in 93% of cases [94]. This is consistent with the known sexual transmission of oncogenic HPV. CIN/SIL may progress to cancer or, more often, it will resolve. Thus cofactors are suspected. Smegma, obtained from under the foreskin of human and horse, was shown to be capable of producing cervical cancer in mice in one study [163], but not in another [105]. Differences in exposure time in each study could have contributed to this difference, and followup is needed to confirm whether or not these old studies have any validity.

The large, well-designed, multinational study by the International Agency for Research on Cancer published in the *New England Journal of Medicine* mentioned earlier irrefutably implicated lack of male circumcision in cervical cancer [45]. It involved 1,913 couples in 5 global locations in Europe, Asia, and South America. As stated earlier, penile HPV was found in 19.6% of uncircumcised, but only 5.5% of circumcised men (adjusted OR = 0.37; 95% CI = 0.16–0.85; *P* < .001). Monogamous women whose male partner had had 6 or more sexual partners were over 5.6 times more likely to have cervical cancer if their partner was uncircumcised (OR = 0.18; 95% CI = 0.04–0.89). Male circumcision was also protective in women whose partner had an intermediate sexual behavior risk index (OR = 0.50; 95% CI = 0.27–0.94). In this study, penile HPV infection was associated with a 4-fold increase in the risk of cervical HPV infection in the female partner, and cervical HPV infection was associated with a 77-fold increase in the risk of cervical cancer. In an accompanying editorial it was suggested that “reduction in risk among female partners of circumcised as compared with uncircumcised men may well be more substantial than reported" [164]. 

Genital HPV types are highly infectious and can infect skin throughout the genital region. Skin-to-skin contact that does not extend to actual sexual penetration by an uncircumcised penis could infect women. In the *NEJM* study condom use provided only a slight protective effect—the odds ratio between condom users and nonusers (0.83) was not statistically significant [45]. A study in Seattle of university undergraduates, however, found that HPV incidence in women whose partners always used condoms was 70% less than those whose partners used condoms less than 5% of the time [165]. Squamous intraepithelial lesions were absent in the group with 100% condom use, compared with an incidence of 15 per 100 patient-years in non-users. Interestingly, the uncircumcised men washed their genitals more often after intercourse, but the circumcised men had better penile hygiene when examined by a physician. So why are uncircumcised men more likely to get infected? One suggested reason is that the more delicate, mucosal lining of the foreskin is pulled back fully or partially during intercourse, exposing it to the vaginal secretions of an infected woman. The higher incidence of HPV in uncircumcised men translates into an increased risk of infection to future sexual partners.

An ecological analysis of data from 117 developing countries revealed a cervical cancer incidence of 35 per 100,000 women per year in 51 countries with a low (<20%) circumcision prevalence compared with 20 per 100,000 in 52 countries with a high (>80%) circumcision prevalence (*P* < .001) [166]. Of all factors examined, male circumcision had the strongest association with cervical cancer incidence.

A meta-analysis of 14 studies up until September 2007 (5 in the USA, 2 in Mexico, 2 in Australia, and one each in South Korea, Denmark, England, Kenya, and the multinational study involving Brazil, Spain, Thailand, and The Philippines referred to above) found an OR of 0.75 (95% CI 0.49–1.14) for the association between male circumcision and cervical cancer in monogamous women [67].

A RCT in Rakai, Uganda, studied the female partners of men who underwent circumcision and those of men who remained uncircumcised [167]. Of these women, 84% were monogamous and 97% had had only one sex partner in the previous year. At the 2-year point an as-treated analysis showed that the 544 whose male partner had been circumcised had a lower prevalence of high-risk HPV infection (28%) than the 488 whose male partner was uncircumcised (38%): PRR = 0.75. For low-risk HPV these figures were 35% and 41%, respectively (PRR = 0.83). The prevalence of multiple high-risk HPV was 8.9% and 12.6%, respectively, giving an IRR of 0.71, while that of multiple low-risk HPV was 9.2% and 14.2% (IRR = 0.65). Between enrolment and year 2 the prevalence of high-risk HPV decreased by 7.4%  (*P* = .006) in the women whose male partner had been circumcised, but did not change significantly (+1.6%) in the women whose male partner had remained uncircumcised. The study also found that by 2 years women with circumcised partners had cleared 82% of high-risk HPV acquired during the first year of the trial, compared with 70% for women with uncircumcised partners (*P* = .14). The authors pointed out that the estimated efficacy of male circumcision in prevention of high-risk HPV (28%) could, for a number of reasons, have been an underestimate.

Thus the epidemic of cervical cancer worldwide in women would appear to be facilitated, at least in part, by the lack of circumcision in men. We speculate that in countries that have experienced a downturn in the uptake of neonatal circumcision, as occurred in the USA and to a greater extent in Australia in the late 1970s and 1980s, the incidence of cervical cancer can be expected to increase. This is because these males would now have reached sexual maturity. The higher proportion of uncircumcised men in the male population increases the overall risk to women today, more than would otherwise have been the case if male circumcision prevalence had remained high.

Prophylactic vaccines against HPV 16 and 18 became available for administration to girls prior to sexual activity in 2007. These two HPVs represent 70% of the HPV types found in cervical cancers. They were also the two genotypes that had the highest population prevalence in the past. In 2007, however, the CDC reported that HPV 16 and 18 are now less prevalent, type 16 becoming only the 6th most common and HPV18 now being even less prevalent [168]. Moreover, replacement of types 16 and 18 by other HPV types not included in current vaccines could occur. Other concerns include the very high ongoing costs of vaccination programs, levels of uptake, the possibility of the need for booster doses if efficacy wanes over time, weak cross-genotype immunity, poor efficacy in women with prior HPV 16 and 18 infections, and the false belief that the vaccines protect against all cervical cancer, which may result in fewer women continuing to participate in screening programs or practicing safe sex. 

Various studies have demonstrated increasing infection with genital HPV types at a younger age. In the UK, 5% of girls under 14 had HPV antibodies, indicating current or prior infection [169]. By age 16 this was 12%, by 18 it was 20%, and by age 24 the proportion infected was 45%, with a subsequent decrease thereafter. Oncogenic HPV16 was the most common type. In the USA, 7% of teenagers (ages 12–19) had HPV16 antibodies, rising to 25% for 20–29-year olds [170]. Chlamydia and genital herpes cases are also rising in teenagers in developed countries. 

HPV can be transmitted to the mouth during oral sex and is an independent risk factor for some oropharyngeal cancers [171]. 

It should be noted that there might be as many as 200 types of HPV, up to 50 of which have been described in the anogenital region. Most of these range from uncommon to extremely rare. The number of HPV types relevant to screening for cervical cancer risk in the population is approximately 20. Ideally, many expect that vaccination against the most common types (HPV 16 and 18) could prevent two-thirds of cervical cancers. A randomized, placebo-controlled, double-blind trial involving 5,455 women aged 16–24 years found that vaccination reduced the rate of cervical lesions by only 20% [172]. The study lasted only 3 years however. One study found HPV vaccination to not be cost-effective, even under favourable assumptions for vaccination programs [173]. Yet a subsequent review of cost-effectiveness studies concluded that vaccination of girls against HPV will be cost-effective [174]. At an uptake of 80% in 12 year-old girls, HPV vaccines could reduce cervical cancer by 38–82% over 60 years of an ongoing vaccination programme, should vaccine protection last 20 years [175]. Vaccination of boys has, however, been found to not be cost-effective [174, 175].

Complete elimination of HPV 16 and 18 from the population by vaccination might, under optimal conditions of uptake and efficacy, take 20–30 years or more. In the meantime at the population level, other oncogenic HPV types not included in vaccines might take over and replace these two types of HPV [176]. Participation in vaccination programs has been impeded by the “religious right” who have expressed concerns that vaccination will increase promiscuity. Moreover, like the anticircumcision movement, vigorous anti-immunization lobby groups also exist. Most of the adverse events that have received publicity in the news media were not related to the vaccine in the first place and would be seen in any large-scale vaccination program by pure coincidence. One exception might be Guillain-Barre syndrome, which is known to be an uncommon adverse consequence of vaccination in a minority of individuals. The two HPV vaccines currently on the market appear to be at least as safe as any other vaccine, although HPV vaccines can increase tumour invasiveness if a tumour is present [177].

Given the high cost of vaccinating all girls compared with the lesser cost and proven protective effect of universal male circumcision against a raft of other conditions and diseases in men [178, 179], the latter would appear to be a better investment. In women it would help reduce the burden of cervical cancer, and, more recently, the possiblity of a small proportion of breast cancers [180–186], but also herpes simplex virus type 2 [55, 187, 188], less assuredly* Chlamydia trachomatis* [189, 190], then *Trichomonas vaginalis*, bacterial vaginosis, genital ulceration [179, 191–193], and bacterial vaginosis associated with CIN/SIL [194].

## 10. Conclusion

There is now overwhelming evidence that male circumcision affords very strong protection against penile cancer. Unlike the many other conditions that affect up to half of uncircumcised males over their lifetime [178], penile cancer affects only about 0.1% of uncircumcised men. Although rare, its devastating effect and poor prognosis in those affected, and impact on their families, should not be downplayed, especially in the developing countries where penile cancer rates are highest and treatment options are limited. Very importantly, given its role in protecting against cervical cancer, HIV, other STIs and medical conditions, programs aimed at increasing infant male circumcision now would be an excellent investment of public monies for the long run. They would complement the enormously expensive vaccination programs targeting two of the over 15 high-risk HPV types that cause cervical cancer. This strategy would add the many other benefits of male circumcision to the equation [178, 179].

## Figures and Tables

**Figure 4:**
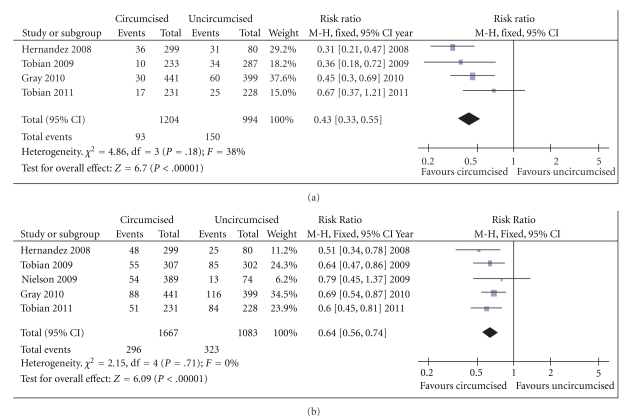
Fixed effects forest plot models of the unadjusted risk ratios for (a) any high-risk HPV and for (b) multiple high-risk HPV infections detected at the coronal sulcus/glans in circumcised and uncircumcised men. The data are derived from both observational studies and randomized trials, and include HPV prevalence and incidence estimates. The unadjusted risk ratios differ from the adjusted odds ratios reported in some studies, because the odds ratio does not approximate to the risk ratio with common disease outcomes such as HPV, and because adjustment for risk behaviors may affect estimates. Nevertheless, the findings are in general consistent across studies as indicated by the nonsignificant tests for heterogeneity and suggest that, in aggregate, circumcision may reduce any high-risk HPV infection by approximately 36%, and multiple high risk HPV infections by 57%.

**Table:** (a) Castellsagué et al. (2002) [45]: Spain, Columbia, Brazil, Thailand, Philippines; 26% were aged ≤37, 57% were 38–56, 25% were ≥57 years

Site	HPV	Circumcised	Uncircumcised	
		(*n* = 370)	(*n* = 1543)	AOR (95% CI)
*Glans, coronal sulcus*				
	Any HPV	5.5%	19.6%	0.37 (0.16–0.85)

**Table:** (b) Svare et al. (2002) [54]: Copenhagen, Sweden, STI clinic

Site	HPV	Circumcised	Uncircumcised	
		(*n* = 22)	(*n* = 112)	AOR (95% CI)
*Glans, coronal sulcus, *				
*Shaft, scrotum*				
	Any HPV	9%	21%	0.20 (0.06–0.60)

**Table:** (c) Baldwin et al. (2004) [44]: Tucson, Arizona, STI clinic, 42% white (non-Hispanic), 39% Hispanic, 19% indigenous, Pacific Islander or Asian, age 18–70, 90% heterosexual, 82% single, condom users had 79% lower high-risk HPV and 42% higher low-risk HPV

Site	HPV	Circumcised	Uncircumcised	
		(*n* = 232)	(*n* = 89)	AOR (95% CI)
*Glans, coronal sulcus,*				
*urethral meatus*				
	Any HPV	19.8%	41.1%	0.34 (0.20–0.57)
	High-risk	7.8%	18.8%	0.44 (0.22–0.90)
	Low-risk	12.1%	22.3%	0.44 (0.23–0.81)

**Table:** (d) Weaver et al. (2004) [58]: Seattle, university students, aged 18–25, 81% white, 6% Asian, 3% African American, 2% Latino, 8% other, 97%  *β*-globin DNA-positive

Site	HPV	Circumcised	Uncircumcised	
		(*n* = 233)	(*n* = 84)	OR (95% CI)
*Glans*	Any HPV	17%	32%	Not shown
*All sites*	Any HPV	31%	29%	0.95 (0.50–1.79; NS)

**Table:** (e) Shin et al. (2004) [52]: South Korea, university students, 46% had ≥4 sex partners

Site	HPV	Circumcised	Uncircumcised	
		(*n* = 296)	(*n* = 40)	OR (95% CI)
*Coronal sulcus, meatus,*				
*shaft, scrotum*				
	Any HPV	7.0%	8.9%	1.8 (0.4–8.2; NS)

**Table:** (f) Lajous et al. (2005) [48]: Mexico City, soldiers, age 16–40 (av. 23), average 3 sex partners. condom use with prostitutes did not affect HPV prevalence

Site	HPV	Circumcised	Uncircumcised	
		(*n* = 95)	(*n* = 830)	AOR (95% CI)
*Coronal sulcus, meatus,*				
*shaft, scrotum*				
	Any HPV	29.5%	44.0%	0.48 (0.30–0.77)

**Table:** (g) Vaccarella et al. (2006) [57]: Mexico, 27 public vasectomy clinics in 14 states, average age 34 years, HPV was 60% less for condom users with regular partners and 90% less with sex workers, high-risk and low-risk HPV stated as similar within each of circumcised and uncircumcised

Site	HPV	Circumcised	Uncircumcised	
		(*n* = 247)	(*n* = 532)	AOR (95% CI)
Glans/coronal sulcus,				
Meatus, shaft, scrotum				
	Any HPV	2.4%	11.7%	0.20 (0.10–0.40)

**Table:** (h) Partridge et al. (2007) [51]: Seattle, university students, age 18–20, white 85%, Asian/Pacific Islander 8.3%, other 7.1%, unmarried

Site	HPV	Circumcised	Uncircumcised	
		(*n* = 184)	(*n* = 56)	HR (95% CI)
*Glans, shaft, scrotum*	1.2/100PY	1.7/100PY	1.1 (0.6–2.0; NS)	

**Table:** (i) Hernandez et al. (2008) [59]: Hawaii, university population, most white, single, average age 29, 77% heterosexual, 53% had had ≥6 female sex partners, 50% used condoms, all HIV-negative

Site	HPV	Circumcised	Uncircumcised	
		(*n* = 299)	(*n* = 80)	AOR (95% CI)
*Glans/coronal sulcus*				
	Any HPV	29%	46%	0.51 (0.27–0.97)
	High-risk	16%	31%	0.40 (0.18–0.90)
	Low-risk	22%	39%	0.51 (0.25–1.08)
	Multiple	12%	39%	0.28 (0.12–0.67)
*Shaft*				
	Any HPV	50%	60%	0.63 (0.42–1.22)
	High-risk	34%	38%	0.70 (0.32–1.52)
	Low-risk	45%	56%	0.59 (0.30–1.16)
	Multiple	30%	36%	0.57 (0.26–1.28)
*Urine*				
	Any HPV	8%	16%	0.31 (0.08–1.16)
	High-risk	1%	3%	0.18 (0.004–7.69)
	Low-risk	7%	16%	0.28 (0.07–1.10)
	Multiple	1%	0%	—
*Semen*				
	Any HPV	6%	5%	1.09 (0.17–7.14)
	High-risk	2%	0%	—
	Low-risk	6%	5%	0.86 (0.13–5.88)
	Multiple	1%	0%	—
*Scrotum*				
	Any HPV	40%	40%	0.82 (0.43–25.0)
	High-risk	20%	20%	0.69 (0.33–2.38)
	Low-risk	33%	35%	0.69 (0.33–1.43)
	Multiple	14%	19%	0.53 (0.21–1.33)
External penis				
	Any HPV	57%	67%	0.58 (0.30–1.14)
	High-risk	25%	23%	0.82 (0.28–2.38)
	Low-risk	30%	36%	0.61 (0.25–1.47)
	Any	20%	23%	0.52 (0.17–1.56)
*Any site*				
	Any HPV	78%	83%	0.49 (0.19–1.28)
	High-risk	55%	58%	0.38 (0.11–1.28)
	Low-risk	61%	67%	0.42 (0.14–1.25)
	Multiple	39%	41%	0.35 (0.09–1.43)

**Table:** (j) Nielson et al. (2009) [49]: Tucson and Tampa, aged 18–40, circumcised participants: white 76%, Indigenous 6%, black 1%, Asian/Pacific Islander 2%, other 4%; >6 sex partners 65%, condom use ≤ half = 56%, sex with partner with abnormal pap smear 26% for circumcised, 11% for uncircumcised

Site	HPV	Circumcised	Uncircumcised	
		(*n* = 389)	(*n* = 74)	AOR (95% CI)
*Glans/coronal sulcus*				
	Any HPV	29.8%	35.2%	0.44 (0.23–0.82)
	High-risk	13.9%	18.3%	0.47 (0.22–0.99)
	Low-risk	15.8%	16.9%	0.62 (0.29–1.29)
*Shaft*				
	Any HPV	40.2%	40.9%	0.53 (0.28–0.99)
	High-risk	21.2%	25.4%	0.50 (0.25–1.00)
	Low-risk	19.1%	15.9%	0.85 (0.40–1.80)
*Scrotum*				
	Any HPV	25.9%	24.3%	0.73 (0.37–1.44)
	High-risk	12.9%	12.9%	0.68 (0.29–2.06)
	Low-risk	12.9%	11.4%	0.86 (0.36–2.06)
*Urethra*				
	Any HPV	7.8%	14.9%	0.17 (0.05–0.56)
	High-risk	3.9%	2.1%	1.24 (0.14–10.8)
	Low-risk	3.9%	12.8%	0.04 (0.01–0.23)
*Semen*				
	Any HPV	4.2%	7.1%	0.48 (0.12–1.96)
	High-risk	3.1%	3.6%	0.41 (0.10–2.78)
	Low-risk	1.1%	3.6%	0.41 (0.03–5.07)
*Any site*				
	Any HPV	51.2%	51.4%	0.53 (0.28–0.99)
	High-risk	28.8%	31.2%	0.56 (0.30–1.06)
	Low-risk	22.4%	20.3%	0.84 (0.43–1.67)

**Table:** (k) Giuliano et al. (2009) [46]: USA (34%), Mexico (32%), Brazil (35%); age 18–70 (av. 32), 66% had >1 sex partner in past 3 months, 9% had had sex with male, condom use: always 20%, sometimes 32%. The respective OR became 0.70 (0.52–0.94), 0.70 (0.50–0.97), and 0.63 (0.42–0.93) after multivariate analysis

Site	HPV	Circumcised	Uncircumcised	
		(*n* = 590)	(*n* = 398)	OR (95% CI)
*Coronal sulcus, shaft, under foreskin, scrotum (all *β*-globin DNA-positive)*				
	Any HPV	54.8%	62.2%	0.97 (0.68–1.39)
	High-risk	41.8%	49.2%	0.93 (0.63–1.33)
	Low-risk	33.1%	40.4%	1.15 (0.74–1.79)

**Table:** (l) Auvert et al. (2009) [43]: RCT, South Africa, Black, age 18–24, average 4 lifetime sex partners, consistent condom use 25%, 5% HIV-positive

Site	HPV	Circumcised	Uncircumcised	
		(643)	(621)	PRR (95% CI)
*Urethra*				
	High-risk	14.0%	23.2%	0.60 (0.46–0.79)
	Multiple high-risk	4.2%	9.9%	0.43 (0.28–0.66)

**Table:** (m) Tobian et al. (2009) [55]: Rakai 1 RCT, Kenya; age 15–49 years; only *β*-globin positive samples

Site	HPV	Circumcised	Uncircumcised	
		(*n* = 307)	(*n* = 302)	RR (95% CI)
*Glans/coronal sulcus*				
	All HPV	35.6%	51.2%	0.70 (0.53–0.91)
	High-risk	18.0%	27.9%	0.65 (0.46–0.90)
	Low-risk	26.2%	39.4%	0.66 (0.49–0.91)
	Multiple	4.3%	12.2%	0.35 (0.17–0.71)

**Table:** (n) Gray et al. (2010) [47]: Rakai, Uganda; RCT; age 15–24 (22%), 25–35 (51%), >35 (26%); condom use (35%), >1 sex partners 42%; HPV at enrolment (39%); data for 24 months: after circumcision

Site	HPV	Circumcised	Uncircumcised	
		(*n* = 441)	(*n* = 399)	IRR (95% CI)
*Glans/coronal sulcus*				
	Any high-risk HPV	19.7%	29.4%	0.67 (0.50–0.90)
	Single new high-risk HPV	12.9%	15.6%	0.89 (0.60–1.30)
	Multiple new high-risk HPV	6.7%	14.8%	0.45 (0.28–0.73)
	HPV16	3.6/100PYs	4.8/100PYs	0.75 (0.38–1.51)
	HPV18	1.6/100PYs	5.3/100PYs	0.30 (0.12–0.75)
	HPV31	1.6/100PYs	2.2/100PYs	0.74 (0.27–2.05)
	HPV33	0.5/100PYs	3.1/100PYs	0.17 (0.04–0.76)
	HPV35	1.9/100PYs	3.7/100PYs	0.50 (0.21–1.21)
	(Condom use)	22/100PYs	32/100PYs	0.68 (0.43–1.09)

**Table:** (o) Tobian et al. (2011) [56]: Rakai, rural Uganda, RCT, age 15–49, consistent condom use 16%, HPV test at 12 months after circumcision, only *β*-globin DNA positive samples included, high-risk HPV significantly higher on coronal sulcus than on shaft

Site	HPV	Circumcised	Uncircumcised	
		(*n* = 231)	(*n* = 228)	APR (95% CI)
*Coronal sulcus*				
	Any high-risk HPV	21.5%	36.3%	0.57 (0.39–0.84)
	Multiple high-risk	7.4%	10.5%	0.71 (0.33–1.52)
Shaft				
	Any high-risk HPV	15.5%	23.8%	0.66 (0.39–1.12)
	Multiple high-risk HPV	1.7%	3.8%	0.45 (0.09–2.27)

OR: odds ratio; AOR: adjusted odds ratio; NS: not significant; RR: risk ratio; PPR: prevalence risk ratio; HR: hazard ratio, 100PY: 100 person years.

**Table 2:** Association between phimosis and penile cancer.

Study [ref.]	*n*/N*	OR (95% CI)	Type
Brinton et al. (1991) [99]	44/111	37.2 (11.9–116)	IPC
Tsen et al. (2001) [16]	50/150	1.7 (0.32–7.8)^†^	CIS
Tsen et al. (2001) [16]	50/150	16 (4.5–57)^†^	IPC
Daling et al. (2005) [36]	33/308	3.8 (1.4–10.1)^†^	CIS
Daling et al. (2005) [36]	38/313	11.4 (5.0–25.9)^†^	IPC
Velazquez et al. (2003) [98]	23/238	14.5 (5.5–38.4)	Not stated
Harish & Ravi (1995) [100]	503/1006	6.97 (4.3–11.3)^†^	Not stated
Hellberg et al. (1987) [101]	217/414	64.6 (30.9–135)	Not stated

Meta-analysis (random effects): OR = 12.1 (95% CI = 5.57–26.2)			

*Total cases/total participants; ^†^Adjusted odds ratio presented in original study.

IPC, invasive penile carcinoma; CIS, carcinoma *in situ*.

**Table 3:** Association between smegma and penile cancer.

Study [ref.]	*n*/N*	OR (95% CI)	Type
Maden et al. (1993) [3]	80/268	2.1 (1.2–3.8)^†^	IPC+CIS
Brinton et al. (1991) [99]	30/97	11 (3.68–32.6)	IPC
Daling et al. (2005) [36]	32/308	1.4 (0.3–6.9)^†^	CIS
Daling et al. (2005) [36]	38/314	2.4 (0.7–8)^†^	IPC

Meta-analysis (random effects): OR = 3.04 (95% CI = 1.29–7.16)			

*Total cases/total participants; ^†^Adjusted odds ratio presented in original study.

IPC, invasive penile carcinoma; CIS, carcinoma *in situ*.

**Table 4:** Association between balanitis and penile cancer.

Study [ref.]	*n*/N*	OR (95% CI)	Type
Maden et al. (1993) [3]	100/199	1.3 (0.5–3.6)^†^	IPC+CIS
Daling et al. (2005) [36]	74/743	3.5 (1.2–10.3)^†^	CIS
Daling et al. (2005) [36]	62/731	3.9 (1.3–11.7)^†^	IPC
Hellberg et al. (1987) [101]	207/400	9.49 (5.24–17.2)	Not stated

Meta-analysis (random effects): OR = 3.82 (95% CI = 1.61–9.06)			

*Total cases/total participants, ^†^Adjusted odds ratio presented in original study.

IPC, invasive penile carcinoma; CIS, carcinoma *in situ*.
